# Supplementary material for: Patterns of Intron Gain and Loss in Fungi
Source: PLoS Biol. 2004 Nov 30;2(12):e422. doi: 10.1371/journal.pbio.0020422 (PMC532390; doi:10.1371/journal.pbio.0020422)
Supplement: Table S1 — Also available at http://genes.mit.edu/NielsenEtAl/. (4.3 MB ZIP). [file pbio.0020422.st001.zip › NielsenEtAl/html/563.html]

AN0705.1.NCU03575.1.MG03220.1.FG10722.1


```
 CLUSTAL W (1.82) Multiple Sequence Alignments - Introns Inserted


Sequence 1: NCU03575.1	1079 aa
Sequence 2: MG03220.1	1077 aa
Sequence 3: FG10722.1	1080 aa
Sequence 4: AN0705.1	1077 aa
Alignment Length: 1094 aa
Number Identitical Residues: 700 aa
Alignment Score (without introns) 32718


MG03220.1 	MSISFPKEEEAVIARWREINAFERQ0VELSQGRPLYTFYDGPPFATGLPHYGHLLASTIK
NCU03575.1	MSIDFPKEEENTIQRWREIKAFETQ~VELSKGNPLYTFYDGPPFATGLPHYGHLLASTIK
FG10722.1 	MSIDFPKEEEVVLQRWREIDAFLRQ0VELSEGRPRYTFYDGPPFATGLPHYGHLLTSTIK
AN0705.1  	MSIDFPKEEETILKRWKEINAFQRQ~VELSKGRKPYTFYDGPPFATGLPHYGHLLASTIK
          	***.******  : **:**.**  * ****:*.  ********************:****

MG03220.1 	DIIPRYWSMKGYHVERRFGWDTHGLPIEHEIDKKHGISGKAAVEKFGLANYNEECRSIVM
NCU03575.1	DIIPRYWSMKGFHVERRFGWDTHGLPIEHEIDKKLGISGKAAVMEIGLENYNAECRSIVM
FG10722.1 	DVIPRYWSMKGFHVERRFGWDTHGLPIEHEIDKKLGISGKAAVMELGIAKYNEECRSIVM
AN0705.1  	DIIPRYWSMKGHYVERRFGWDTHGVPIEYEIDKKLGMSGLEAVEKLGIEKYNEECRAIVM
          	*:*********.:***********:***:***** *:**  ** ::*: :** ***:***

MG03220.1 	RYREEWRHTIERLGRWIDFDNDYK0---------~TMDPKFMESEWWVFKQLFEKGQVYQ
NCU03575.1	RYREEWRHTVERLGRWIDFDNDYK~---------~TMDPTFMESEWWVFKQLFDKGQVYR
FG10722.1 	RYAKEWRVTVERLGRWIDFDNDYK~VKKPTTICI0TMDPSFMESEWWVFKQLFDKDQVYQ
AN0705.1  	RFASEWRETIERLGRWIDFDNDYK0---------~TMNTSFMESVWWVFKQLFDKGLVYK
          	*: .*** *:**************           **:..**** ********:*. **:

MG03220.1 	GFRVMPYSTVLTTALSNFEANQNYQDVTDPAVVVSFPLKSDPETSLLAWTTTPWTLPSNL
NCU03575.1	GYRVMPYSTALTTALSNFEANQNYQDVTDPAVVVAFPLIEDPNTCLLAWTTTPWTLPSHT
FG10722.1 	GHRVMPYSTVLTTALSNFEANQNYQDVTDPAVVVTFPLVDDPDVNLLAWTTTPWTLPSHL
AN0705.1  	GFRVMPYSTALNTPLSNFEAQQNYKDVQDPAIVVTFPLLDDPETCLLAWTTTPWTLPSNT
          	*.*******.*.*.******:***:** ***:**:*** .**:. *************: 

MG03220.1 	GLAAHPDFEYVKIRDEKSGKNYILLEKLMGTLYKDPKKAKFQILQKIKGKDMLGWEYEPL
NCU03575.1	GLAAHPDFEYVKIADEKSGKNYILLEKLLTTLYKDPKKAKFKVLEKIKGKDMLGWKYEPP
FG10722.1 	GLAAHPDFEYVKILDEKSGKIYILLEKLLGTLYKDPKKAKFKIVEKILGKDMLGWKYTPP
AN0705.1  	ALAVHPDFEYIKIFDEASGKHYILLESLLRTIYKDPKKAKFKIVSKFKGSEMKDWKYQPL
          	.**.******:** ** *** *****.*: *:*********:::.*: *.:* .*:* * 

MG03220.1 	FDYFYEEFKDVGFRVLNATYVTDDGGVGIVHQAPAFGEDDYNVASEAGIINENRPPADPV
NCU03575.1	FNYFYDEYKDVAFKVLNATYVTDDSGTGIVHQAPAFGEDDYNVAVEAGIVTEKRPPPDPV
FG10722.1 	FNYFYDEFKDVAFKVLNATYVTDDSGVGIVHQAPAFGEDDYNVAVAAGIVTENRSPPDPV
AN0705.1  	FNYFYETFKDHGYRVLNATYVTADDGTGIVHQAPSYGEDDYKVGVEGGVIDETRPPPNPV
          	*:***: :** .::******** *.*.*******::*****:*.  .*:: *.*.*.:**

MG03220.1 	NETGHFTDKVRDFAGMHVKEADKHIIKYLKGTGRLVADSQLKHSYPMCPRSDTPLIYRAV
NCU03575.1	NETGHYTDRVSDFAGMHVKEADKHIIKHLKNSGKLVVESQLKHSYPMCYRSDTPLIYKAV
FG10722.1 	NDTGHFTDRVSDFKGMHVKEADKHIIKYLKNAGRIANESQLKHSYPMCPRSDTPLIYRAV
AN0705.1  	DDMGCFTSEVPEFQGQHVKAADKAIIKHLKGTGRLLVDSQITHSYPFCWRSDTPLIYRAV
          	:: * :*..* :* * *** *** ***:**.:*::  :**:.****:* ********:**

MG03220.1 	PSWFVRIPEIVPKMLENIETQHWVPSFVKEKRFASWIANARDWNVSRNRYWGTPIPLWVS
NCU03575.1	PSWFIRIPDIIPQMLKNIEGSHWVPSFVKEKRFASWIANARDWNVSRNRYWGTPIPLWMS
FG10722.1 	PSWFIRIPDIIPDMLKNIEETRWVPSFVKEKRFASWIANARDWNVSRNRYWGTPIPLWVS
AN0705.1  	PSWFVKIQPIIPKMLEGIEDSHWVPSAVKDKRFASWIQNARDWNISRNRFWGTPLPLWAN
          	****::*  *:*.**:.**  :**** **:******* ******:****:****:*** .

MG03220.1 	DDLEERVCVGSIEELKKLSGYEGEITDLHRHHIDHITIPSKQGKGVLKRIEEVFDCWFES
NCU03575.1	DDGEEIVCIGSIEELKQLSGYEGEITDLHRDKIDHITIPSKQGKGVLKRAEEVFDCWFES
FG10722.1 	EDLEERVCVGSVQELRDLSGYEGDLSDLHRDKVDHITIPSKMGKGQLKRIEEVFDCWFES
AN0705.1  	EDFSEVVAVGSIQELKELSGYEGEITDIHRDKVDKITIPSKKGNGVLRRVSEVFDCWFES
          	:* .* *.:**::**:.******:::*:**.::*:****** *:* *:* .*********

MG03220.1 	GSMPYASQHYPFENVEKFEKSFPGDFIAEGLDQTRGWFYTMLVLGTHLFGIAPFKNCVVN
NCU03575.1	GSMPYASQHYPFENVEKFEKSFPGDFIAEGLDQTRGWFYTLLVLGTHLFGVSPFKNCVVN
FG10722.1 	GSMPYASQHYPFENVEKFEQSFPGNFIAEGLDQTRGWFYTLTVLGTHLFGKSPFKNCVVN
AN0705.1  	GSMPYAQQHYPFENKEQFEKSFPGDFIAEGLDQTRGWFYTLTVLGTHLFGKLPFKNCVVN
          	******.******* *:**:****:***************: ********  ********

MG03220.1 	GIVLAEDGKKMSKRLKNYPAPDIVMDKYGSDALRLYLINSPVVRAEPLRFKETGVKEVIQ
NCU03575.1	GIVLAEDGKKMSKRLKNYPDPTLVMNKYGSDALRLYLINSPVVRAEPLRFKEAGVKEVVA
FG10722.1 	GIVLAEDGKKMSKRLKNYPDPSIIMSKYGSDALRLYLINSPVVRAEPLRFKESGVKEVVQ
AN0705.1  	GIVLAEDGKKMSKRLKNYPDPSLIMDRYGSDALRLYLINSPVVRAEPLRFKESGVKEIVA
          	******************* * ::*.:*************************:****:: 

MG03220.1 	RVLLPFWNSYKFFEGQVALLKKAEEKDYMWNPTAESSNENVMDRWILASCQSLLEFVNEE
NCU03575.1	KVLLPLWNSYKFFEGQVALLKKVENVDYVFDPKMESSNENVMDKWILASCQSLLEFVNEE
FG10722.1 	KVLLPLWNSYKFFEGQVALLKKVENVDFVWDPKLEATNTNVMDRWILASCQSLLAFVNQE
AN0705.1  	KVLLPLWNSYKFFEGQAALLKKTAGIDFMWDPKVEATNTNVMDRWILASCQSLLKFVNQE
          	:****:**********.*****.   *::::*. *::* ****:********** ***:*

MG03220.1 	MRH1YRLYT~VVPRLLGLIDNTTNWYIRFNRKRLKGENGVEDTVHALNTLFEVLFTLCKG
NCU03575.1	MKG1YRLYT~VVPRLLELIDNTTNWYIRFNRKRLKGENGLNDTLHALNTLFEVLFTLCRG
FG10722.1 	MEA1YRLYT0VVPRLLGLIDNTTNWYIRFNRKRLKGENGLDDTLHALNALFEVLFTLCRG
AN0705.1  	MAG~YRLYT~VVPRLLGLIENTTNWYIRFNRKRLKGENGVNDTLHALNTLFEVLYTLVRG
          	*   ***** ****** **:*******************::**:****:*****:** :*

MG03220.1 	LAPFTPFITETIYQKLAPFIPKDLPKELQSSDMRSVHFLPFPTVRQELFDPVVERKVGRM
NCU03575.1	LAPFTPFLTDTIYLKLLPHIP----KELQAKDPRSVHFLPFPEVRKELYNPDVERRVQRM
FG10722.1 	LAPFTPFLTDNIYLKLLPHIP----KELQGDDFRSVHFLPFPDVRQELFDEEVERRVGRM
AN0705.1  	LAPFTPFLTDNIYGRLLPHIP----EAIRGEDSRSVHFLPFPEVREELFDEVVERRVARM
          	*******:*:.** :* *.**    : ::..* ********* **:**::  ***:* **

MG03220.1 	QKVIELARVSRERRAIGLKTPLKTLIVIHHSQEYLDDVKSLQGYISEELNIRDLVLTSDE
NCU03575.1	QKVIELARYSREKRSVGLKQPLKTLVVIHHDEQYLADIESLKGYITEELNVRDLVLTSDE
FG10722.1 	QRVIELARVSRERRTIGLKQPLKTLVVLHSDPQYLEDVKSLKNYISEELNVQDLVLSSDE
AN0705.1  	QKVIEMARVSRERRSLGLKTPLKSLVVIHQDQQYLDDVKSLEGYILEEINIQELILSTDE
          	*:***:** ***:*::*** ***:*:*:* . :** *::**:.** **:*:::*:*::**

MG03220.1 	AKYGVQYSATADWPVLGKKLKKDMAKVKKALPLLQSEQVHAYVQQGTIEVDGIKLDAGDL
NCU03575.1	AKHGVQYSVSADWPVLGKKLKKDMGRVKKALPSVTSDEAHAYTQTGKLVVDGITLEAGDL
FG10722.1 	SKYNVQYSVTADWPVLGKKLKKDMARVKKGLPLLTSDQVKGYLQDKHIDVDGIRLEEGDL
AN0705.1  	EKYNVQYSVSADWPTLGKKLKKDAQKVKKALPSLTSDDVKKFVAEKTILVDGIELVEGDL
          	 *:.****.:****.********  :***.** : *::.: :     : **** *  ***

MG03220.1 	VVKRGLSPAADGSSQYLETNTDNDVLIILDCELHADLADEGLAREIINRVQRLRKKAGLV
NCU03575.1	VVKRGLK--EDDSSKNLETNTDNDVLTILDVEIYPGLAEEGLAREIINRVQRLRKKAGLQ
FG10722.1 	VVRRGVK--EDDSSKNFETNTDSEVLTILDTEIHPELVAEGLGREIINRVQRLRKKAGLV
AN0705.1  	VVKRGLK--QDASAEDKEPAADADVLTILDVKLYPELAHQGLGREIVNRLQRLRKKAGLV
          	**:**:.   * *::  *. :* :** *** :::. *. :**.***:**:********* 

MG03220.1 	ATDDVRMEYKVLGDPETTGIEKVFQSQSSMLEKALRRP--------MEGEVVDQPEDGVI
NCU03575.1	PTDDIKMEYKVTADPEETGIEKVFDTQMAMIEKALRRPLDKHEVTHVEGKIPAEKEEGII
FG10722.1 	PTDDIKMEYRVVADPEDVGLSGAFKSQTPAFEKALRRP--------LEEASAETQTEGLI
AN0705.1  	PTDDVKMEYVVLSDPDNIGIAEAFNTQASVIEKAVRRPLEKRD--SADGKLPSADEEGMI
          	.***::*** * .**:  *:  .*.:* . :***:*** .. .    :        :*:*

MG03220.1 	AEEMQEVQKATFRLRLLKL
NCU03575.1	MEEEQEVGKATFLLRLLRL
FG10722.1 	AEEEQEVQQATFILRLLKL
AN0705.1  	MQEEQEVQNATFLLRLLKL
          	 :* *** :*** ****:*
```
